# Fibrous Remodeling in Eosinophilic Esophagitis: Clinical Facts and Pathophysiological Uncertainties

**DOI:** 10.3390/ijms25020927

**Published:** 2024-01-11

**Authors:** Laura Arias-González, Leticia Rodríguez-Alcolado, Emilio J. Laserna-Mendieta, Pilar Navarro, Alfredo J. Lucendo, Elena Grueso-Navarro

**Affiliations:** 1 Department of Gastroenterology, Hospital General de Tomelloso, Vereda de Socuéllamos s/n, 13700 Tomelloso, Spain; laura.arias.gonzalez@gmail.com (L.A.-G.); leticia3r@gmail.com (L.R.-A.); ejlaserna@sescam.jccm.es (E.J.L.-M.); mpilar_ns@hotmail.com (P.N.); elenagru4@gmail.com (E.G.-N.); 2 Centro de Investigación Biomédica en Red de Enfermedades Hepáticas y Digestivas (CIBERehd), 28029 Madrid, Spain; 3 Instituto de Investigación Sanitaria de Castilla-La Mancha (IDISCAM), 45071 Toledo, Spain; 4 Instituto de Investigación Sanitaria Princesa, 28006 Madrid, Spain

**Keywords:** eosinophilic esophagitis, transforming growth factor beta, inflammation mediators/immunology, inflammation mediators/metabolism, remodeling

## Abstract

Eosinophilic esophagitis (EoE) is a chronic, progressive, type 2 inflammatory disease with increasing global prevalence. An eosinophil-predominant inflammation that permeates the epithelium and deeper esophageal layers characterizes the disease. Several cytokines, mainly derived from inflammatory T-helper 2 (Th2) cells and epithelial cells, are involved in perpetuating inflammatory responses by increasing surface permeability and promoting tissue remodeling characterized by epithelial–mesenchymal transition (EMT) and collagen deposition. This leads to esophageal strictures and narrow caliber esophagi, which are proportional a patient’s age and untreated disease length. Pathophysiological mechanisms leading to EoE have been described in recent years, and transforming growth factor beta (TGF)-beta have been involved in fibrotic phenomena in EoE. However, evidence on the dependence of these phenomena on TGF-beta is scarce and contradictory. This review provides state-of-the art knowledge on intimate mechanisms of esophageal fibrosis in EoE and its clinical consequences.

## 1. Introduction

Eosinophilic esophagitis (EoE) is a syndrome characterized clinically by symptoms related to esophageal dysfunction and histologically by an eosinophil-predominant inflammatory infiltration limited to the esophagus, for which additional secondary causes of esophageal eosinophilia are excluded [1]. First defined in the 1990s [2,3], EoE was initially described as a particular form of food allergy [4], in which immunoglobulin E (IgE) plays quite a limited role [5]. At first considered a rare condition, the epidemiology of EoE in recent years has risen sharply [6], and currently affects up to 1 in 850 people in developed countries [7,8]. EoE is also emerging in developing countries, with cases being reported on all continents. In the absence of treatment, esophageal inflammation and symptoms tend to persist or even worsen over time [9]. The chronicity of EoE symptoms deteriorates patients’ quality of life [10], while the persistence of eosinophilic inflammation has been linked to structural changes in the esophagus [11], thus indicating a need to treat patients with active disease.

The cumulative literature over the last 3 decades has described the clinical picture of EoE [12] and investigated several therapeutic approaches. These are either based on dietary modifications aimed at identifying and avoiding food triggers, or on drugs with anti-inflammatory effects and consisting of topical corticosteroids and proton pump inhibitors [13]. An increasing number of studies focused on characterizing the disease at the molecular level [14] have also allowed for the development of new therapies, mainly based on monoclonal antibodies that target cytokines involved in the pathophysiology of EoE [15]. However, the pathophysiological processes involving cells and cytokines that contribute to EoE, its regulatory mechanisms and the complex interplay between genes and the environment that triggers it [16] still need to be fully understood [17].

This review aims to provide an overview of the inflammatory phenomena in EoE, its chronicity, progression to esophageal fibrosis, and its clinical consequences and to reveal some pathophysiological issues which have not been fully analyzed until now.

## 2. Immunological Aspects of EoE

EoE is characterized by a specific cytokine secretion pattern that determines it as a type 2 inflammatory disease [18,19]. In healthy conditions, T helper 2 (Th2) cells are primarily important in defense against helminth infections and exposure to venoms [20], but in atopic individuals, they are also involved in different types of allergic diseases including asthma, atopic dermatitis, allergic rhinitis, and food allergies [21]. Thus, a Th2 response is induced in EoE as a particular form of non-IgE-mediated food allergy, and typically associated with increased expression of interleukin (IL)-4, IL-5, IL-13, and eotaxins [22].

IL-5 promotes the differentiation, maturation, and release of eosinophils from the bone marrow [23], enhances eosinophil trafficking to the esophagus [24] and, in murine models of EoE, drives esophageal fibrous remodeling [25]. Among other functions, IL-4 promotes the differentiation of Th2 lymphocytes, the proliferation and differentiation of B lymphocytes, and is a potent inhibitor of apoptosis, thus playing an important role in the development of atopic diseases [26]. Treatment with dupilumab, which blocks IL-4 receptors I and II, has been shown to be effective in several atopies, including asthma and atopic dermatitis, and also improves histological, endoscopic, and EoE symptoms [27,28]. For its part, IL-13, mainly released by esophageal epithelial cells, induces pleiotropic effects in the pathophysiology of EoE. This is due to its ability to selectively reproduce in vitro the esophageal transcriptome characteristic of EoE in epithelial cell cultures [29], and induce the expression and secretion of the eosinophil-activating chemoattractants eotaxin-1/CCL11 and eotaxin-3/CCL26 [30], which then move eosinophils from blood to the esophageal tissues. In fact, eotaxin-3/CCL23 is the most intensively upregulated gene in the esophageal mucosa of EoE patients, compared to controls [31].

In addition, by presiding over the *CAPN14* gene, which codifies for Calpain-14 (CAPN14) esophageal specific protease [32,33], IL-13 downregulates the gene expression of proteins that keep the intercellular spaces sealed. These include filaggrin (FLG) and involucrin (IVL) [34], the adhesion molecule desmoglein-1 (DSG) [35], and tight junction-associated proteins [36]. The alteration of all these proteins leads to epithelial dysfunction, whereby dilated intercellular spaces [37] facilitate luminal antigen penetration into the epithelial layers [38,39] and thus perpetuate inflammatory responses.

The central position of IL-13 in the pathophysiology of EoE at multiple levels is clear, therefore, and is exemplified by its independent ability to produce experimental EoE in mice when intratracheally instilled [40].

IL-13, together with IL-4 are synergistically involved in activating fibroblasts to secrete enhanced amounts of extracellular matrix (ECM) [41,42]—a complex three-dimensional network of interlaced fibrillar proteins, multiple matrix protein macromolecules, proteoglycans, anchored growth factors, and other bioactive components [43]. As a consequence of sustained inflammatory phenomena in the esophageal mucosa, fibrous remodeling is triggered in EoE in a similar way to other type 2 inflammatory conditions [44,45].

Infiltration by eosinophils, as the most significant cell in the inflammatory infiltrate of EoE, is usually found in endoscopic biopsies taken from the epithelial surface. Here, eosinophils frequently tend to aggregate forming microabsceses [46]. However, evidence from surgical specimens also demonstrated eosinophilic infiltration and fibrous remodeling in deep esophageal layers. Eosinophils permeating the submucosal and muscle layers and the neuronal plexus promote fibrous remodeling, intense collagen deposition, and smooth muscle hypertrophy [47,48], all of which contributed to altering the mechanical properties of the esophageal wall and to reducing esophageal distensibility in EoE patients [49]. Murine models also demonstrated transmural inflammatory and fibrotic involvement in experimental EoE [25], the same as in the pathophysiology of bronchial asthma [50].

## 3. Inflammation to Fibrosis in EoE: A Dynamic Process

Inflammation and fibrosis are two inter-related conditions with many overlapping mechanisms [51]. Sustained inflammation may result in fibrosis when it exceeds the normal wound-healing response to injury [52] in a highly orchestrated process, with common pathways that occur in many different tissues. Fibrogenesis results from defined sequences of molecular signals and cellular response mechanisms that provoke tissue inflammation, macrophage activation, and immune cell infiltration. The release of soluble mediators by the tissue (including alarmins, cytokines and chemokines) leads to the local activation of collagen-producing mesenchymal cells, (the composition of ECM varies according to specific tissues [53]) and provides a distinctive cell microenvironment for the various tissue compartments [54]. As a result, local activation of fibroblasts, recruitment of fibroblast precursors, and the transition of various cell types into myofibroblasts lead to an excessive deposition of ECM, which replaces healthy parenchymal tissue with collagen-rich ECM components, thus resulting in a loss of the normal function of affected organs [55].

EoE is characterized by an abnormal exacerbated response to harmless antigens (mainly derived from dietary components). Early-life factors have been involved in the origin of the immune imbalance that converts the innate esophageal surveillance system into a reactive one in susceptible individuals [56]. These are common in other allergic diseases [57], and usually involve changes in the abundance or composition of the esophageal microbiome [58] in adaptive responses, with epithelial cells releasing alarmins such as IL-25, IL-33, and thymic stromal lymphopoietin (TSLP) [59]. TSLP is a relevant player in EoE [59] and other type 2 inflammatory and allergic diseases. It activates antigen-presenting cells, including food antigen-presenting dendritic cells in the esophageal mucosa, and promotes T cells maturation and polarization to Th2 cells [60]. These Th2 cells are also involved in the repair of tissues that are damaged by parasitic infections, and in allergic reactions, and activate macrophages and epithelial cells to enhance the production of ECM, which is crucial for tissue repair. Th2 cells produce the so-called Th2 cytokines IL-4, IL-5, and IL-13.

Tissue remodeling is a dynamic process that combines different mechanisms such as hyperplasia of the epithelium, subepithelial fibrosis, epithelial to mesenchymal transdifferentiation (EMT), angiogenesis, and hypertrophy of esophageal smooth muscle [61]. An EMT is a biological process that allows an epithelial cell, which normally interacts with the basement membrane via its basal surface, to interact with myofibroblasts, undifferentiated mesenchymal cell phenotypes, which achieve migratory capacity, invasiveness, elevated resistance to apoptosis, and greatly increased production of ECM components [62]. EMT contributes to fibrosis in EoE (as it is present in the esophageal tissue of patients with EoE), correlates with inflammatory activity measured as eosinophil density and reverses with treatments that decrease inflammation [41,63].

During the initial inflammatory phase, cytokines produced by Th2 cells target a wide variety of immune and non-immune cells, including fibroblasts, epithelial cells, macrophages, and endothelial cells, which act directly or indirectly to repair injured tissues [45]. In the process of tissue formation, the pro-inflammatory signals weaken, and cell proliferation is initiated by growth factors, such as transforming growth factor-β (TGF-β) and basic fibroblast growth factor (FGF-2). Both promote the induction of fibroblasts proliferation simultaneously, activating macrophages to remove apoptotic cell debris [64]. In particular, macrophages that receive type 2 cytokines (also referred as to alternatively activated or M2 macrophages) are involved in tissue repair in some organs, including the liver, central nervous system, heart, skeletal muscle, and lungs [65]. Interestingly, M2 macrophages activated by IL-4 also contribute to slow the progression of fibrosis [66] in schistosomiasis-induced liver fibrosis. Therefore, macrophages play an essential role in regulating tissue regeneration. They can produce reactive oxygen species that worsen tissue damage, and produce a variety of growth factors, such as insulin-like growth factor 1 (IGF-1), vascular endothelial growth factor (VEGF-α), and TGF-β, which regulate epithelial and endothelial cell proliferation, myofibroblast activation, stem and tissue progenitor cell differentiation and angiogenesis [67].

Myofibroblasts develop from EMT or by TGF-β1-dependent differentiation from fibroblast cells [68], and they are typically found in granulation tissue and scar tissue. Upon activation, these cells secrete ECM components (such as collagen), proliferate, migrate, and become contractile. In the particular case of EoE, in vitro studies demonstrated that esophageal epithelial cells stimulated with the profibrotic cytokines tumor necrosis factor-alpha (TNFα), TGFβ, and IL1β acquired a mesenchymal phenotype to become active myofibroblasts [69]. TGFβ stimulation has a robust effect upon epithelial collagen production [42], and under prolonged activation, myofibroblast activity promotes tissue stiffness, collagen deposition, tissue retraction, and the formation of esophageal strictures, which aggravate dysphagia and cause food impaction.

## 4. Clinical Consequences of Fibrous Remodeling in EoE

The natural history of EoE has been defined as a progressive condition that, in the absence of treatment, tends to evolve from an inflamed to a rigid esophagus [70]. If left untreated, symptoms and esophageal inflammation tend to persist over time, and patients can develop esophageal rings, focal strictures, or a long narrowing in the esophageal caliber [9]. Although not every EoE patient will experience this process [71], the cumulative literature demonstrates a significant prevalence of esophageal strictures and narrow-caliber esophagi in the untreated disease, and this is directly proportional to diagnostic delay. The prevalence of fibrotic features of EoE, based on endoscopic evaluation, increases from 46.5% when the diagnostic delay from symptoms onset is less than 2 years to 87.5% in patients diagnosed after >20 years of experiencing symptoms [72,73]. The chances of finding esophageal strictures in EoE doubles with every 10 years of diagnostic delay [70]. As a consequence, fibrotic phenomena develops proportionally to the duration of the untreated disease and patient’s age [58].

The origin of symptoms in EoE is complex and not fully understood. Displayed symptoms vary according a patient’s age [12], and include abdominal pain, nausea, vomiting, and slow eating as early symptoms in children, and typically dysphagia, food impaction, and heartburn in adults. Differences in disease presentation have been related to progressive fibrous remodeling of the esophagus [74], and also abnormal esophageal motility. This has been documented by manometry records: a variety of ineffective peristalsis patterns have been described in patients with EoE (which reverse after short-term anti-inflammatory treatment), together with dysphagia improvement [75,76,77,78]. Most patients usually develop coping strategies by adapting eating behaviors, or the use of dietary restrictions to manage symptoms and avoid food impactions in particular [79]. Esophageal narrowing and stricture formation under a certain lumen caliber will be a major determinant for food impaction.

The Endoflip™ impedance planimetry system is a novel method with the potential to assess esophageal function overall, which has provided evidence of the fibrotic changes that may appear as a consequence of fibrous remodeling resulting from long-term inflammation in EoE [80]. It allows for the determination of esophageal distensibility and esophageal diameter at the distensibility plateau, thus providing a measure of fibrostenotic severity that can be used to clinically phenotype EoE patients. Esophageal compliance is found to be reduced in EoE patients with a stricturing disease, and Endoflip™ measurements are able to predict the risk of food impaction. However, the potential use of Endoflip™ to assess EoE severity and therapeutic monitoring, as well as its possible superiority to esophageal biopsies to guide EoE treatment, still remains unclear.

## 5. TGF-β1: A Major Mediator of Fibrosis

Chronic inflammatory diseases, including diabetes, cardiovascular diseases, interstitial lung disease, viral and non-viral hepatitis, non-alcoholic steatohepatitis, and immune-related disorders such as scleroderma and inflammatory bowel disease, are all associated with fibrotic tissue responses that determine disease prognosis [81,82,83,84,85,86]. In all these diseases, the fibrosis process is triggered in response to an injury that jeopardizes tissue integrity. It is mediated by pro-fibrotic cytokines, with TGF-β as a key driver [87]. They directly induce differentiation of fibroblasts into collagen-secreting fibroblasts, and their production correlates with dysfunction in affected organs [88].

The TGF-β superfamily consists of more than 40 members of secreted polypeptide growth factors that were identified in 1981 as “transforming”, due to their ability to trigger proliferation in cultured fibroblasts [89]. In particular, TGF-β presents three isoforms characterized by different expression patterns, including TGF-β1 (which is expressed in endothelial, hematopoietic, and connective tissue cells); TGF-β2 (expressed in epithelial and neuronal cells); and TGF-β3 (which is expressed primarily in mesenchymal cells) [90,91]. The three members of the TGF-β family are active as secreted peptides and share similar biological activities in vitro, while eliciting more specific biological responses in vivo [92]. During inflammation and fibrosis, TGF-β1 is the most important member in physiological repair and collagen accumulation, as it regulates accumulation of ECM; it is involved in the modulation of inflammation by suppressing its excess [93]; and regulates essential cellular processes, including proliferation, differentiation, apoptosis, adhesion, and migration in several cell types [94].

Binding of TGF-β1 to its specific receptor activates different signaling pathways, the TGF-β/Smad one being involved in fibrogenesis. Multiple non-canonical (Smad-independent) TGF-β signaling pathways have also been described [95]. The tissue environment greatly determines the effect of TGF-β1 on the same cell and regulates several cellular functions such as actin cytoskeleton changes, tight-junction resolution, and transcriptional regulation [96]. Cellular responses to signaling pathways culminate in the expression of genes involved in tissue repair, with ECM accumulation by proliferation and migration of epithelial and mesenchymal elements being an early effect.

## 6. Contribution of TGF-β1 to Fibrosis in EoE: Distinct Methods and Variable Result

The TGFβ1 signaling pathway has been involved in fibrous remodeling phenomena in several type 2 inflammatory diseases, including bronchial asthma [97,98], atopic dermatitis [99], allergic rhinitis [100], nasal polyposis [101], and idiopathic eosinophilic pneumonia [102], all of them sharing common pathophysiological mechanisms with EoE. The inflammatory and fibrotic phenomena described in these organs are similar to those observed in EoE, and despite the differences in intimate molecular mechanisms, it was assumed that the patterns that occur in asthma [50] could be largely extrapolated to EoE [103].

Several studies, using different experimental approaches, tried to describe the potential involvement of TGF-β1 in the fibrous remodeling that happens in EoE [1] (Table 1). Used strategies ranged from esophageal epithelial cell cultures [104] to murine experimental models of EoE [105,106], while most evaluated biopsies were obtained from EoE patients [103,107,108,109,110,111,112,113,114,115,116]. Tissue results from human EoE were also applied to a murine model [25], and blood samples were exceptionally used [117]. The approach to determine TGF-β1 also varied from one study to another. Some of them attempted to demonstrate the protein mainly by staining TGF-β1-positive cells with immunohistochemistry (IHC) [103,105,108,111,112,114] or immunofluorescence (IF) staining [107,109], while others used ELISA [104,117] or measured the changes in TGF-β1 gene expression in EoE biopsies compared to controls [108,109,110,112,113,114,115,116,117] and the changes after therapy [103,110,111]. As demonstrated, the results of these different approaches have not always been comparable (Table 1).

Immunochemical techniques (IHC [103,105,108,111,112,113,114] and IF [107,109]) on esophageal biopsies were the first ones applied to study the role of TGF-β1 in EoE. Using this semi-quantitative assessment, greater numbers of TGF-β1 positive cells were found in esophageal samples taken from patients with active disease, compared to biopsies obtained after effective anti-inflammatory treatment based on topical budesonide.

In contrast, studies based on quantifying TGF-β1 mRNA expression on esophageal biopsies by reverse transcription polymerase chain reaction (RT-PCR) failed in reproducing the results obtained with IHC or IF [105,110,115,116]: It was immaterial whether the treatment tested was an elimination diet [115] or a topical corticosteroid [110,112], whether it was measured after only 6 weeks [115,116], or after years from the initiation of therapy [112]. Unfortunately, no studies have evaluated whether changes at the gene expression level were reproduced in terms of protein expression, thus preventing definitive conclusions. It also remains unclear whether *TGF-β1* gene expression is increased in patients with EoE compared to healthy controls or those with GERD, as studies are very scarce and show contradictory results [110,116,117,118].

**Figure 1:**
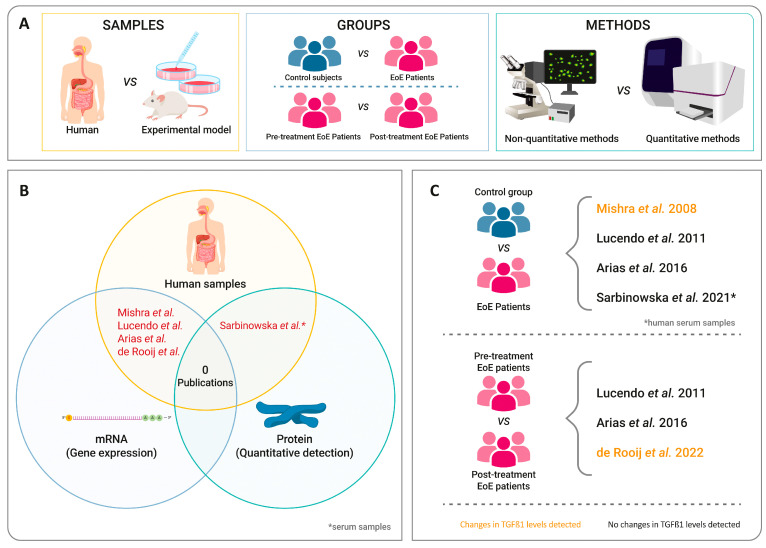
Design of studies reviewed. (**A**) Characteristics of study design. (**B**) Best design classification according to sample type and mRNA or protein of TGF-β1 detected [25,110,115,116,117]. (**C**) Classification of publications reviewed according to the groups of patients compared and the results obtained in the levels of TGF-β1 [25,110,115,116,117].

To provide a deeper insight into the research about TGF-β1 in fibrosis, the study design, the patient or sample groups that were compared, the techniques used to detect TGF-β1 (Figure 1A), and whether the analysis was carried out at the pre-transcriptional or post-translational level were then analyzed. The experimental designs consisted of determining *TGF-β1* mRNA and protein by quantitative methods in human esophageal samples, comparing patients and controls, as well as patients with active EoE versus inactive disease (Figure 1B). Overall, five publications included a more complete study design to determine whether TGF-β1 was involved in EoE fibrosis (Figure 1C), since they were carried out on human samples using quantitative methods.

It seems that the available literature is either inconclusive or inadequate in regard to being able to claim a definitive pro-fibrotic role of TGF-β1 in EoE. This is due to the fact that most studies were performed using cell line cultures [112], or animal models [107,114,119,120], a method which does not necessarily reproduce the pathophysiology of the disease in humans. Where studies were performed on human samples, such as Sarbinowska et al. [117] and Pronio et al. [118], they used an inadequate (serum samples) or limited number of patients, respectively, which increased the risk of biased conclusions. Excluding these research works, only two papers claimed to have found differences in TGF-β1 [25,105], with another two having found no differences [110,115]. However, none of these studies parallelly analyzed TGF-β1 at the protein level (Figure 1C).

## 7. TGFβ Receptor Signaling: The Key of TGF-β1 Effects in EoE?

Recent research has focused attention on the role of TGF-β1 in EoE over its receptor TGFβR1 [121]. There are three receptors specific for the different TGFβ family ligands, which can be distinguished by their structural and functional properties and peptide mapping [122]. Research on murine models has shown that TGFβR participates in the increase in bronchial secretion secondary to rhinovirus infection [123] and in airway smooth muscle cell proliferation [124]. A role of TGFβR 1 variant has been more recently involved in EoE; based on the demonstration, this receptor was essential in maintaining epithelial cell homeostasis to control allergic inflammation in a mouse model of EoE [121]. Knockout mice bearing *TGFβR1* loss-of-function variants developed symptoms, pathological, immunological, and transcriptional changes in the esophagus consistent with human EoE. In particular, the primary defect in epithelial development in knockout mice led to initiate a Th2 inflammatory cascade independent of lymphocytes or allergen exposure. Eosinophilic inflammation in this murine model was restricted to esophageal and gastric mucosa, whilst absent in other organs and tissues. Despite the undoubted value of this observation to advance the knowledge of the potential phenomena leading to fibrosis in EoE, some differences with the disease in humans should be noted, mainly including that mice with experimental EoE developed the disease independently of the exposure to allergens or the presence of lymphocyte infiltrate in the esophageal tissue, which is completely different in the human disease. Exclusive feeding with an elemental diet (completely lacking food antigens) almost universally reverses inflammatory infiltration in EoE patients [4,125], while lymphocytes are part of the cellular infiltrate of the esophagus, and their density varies with eosinophil density [126]. Finally, eosinophilic infiltration of the gastric mucosa is absent in the human form of the disease, but not in this experimental EoE model. The authors justified this finding in that the proximal forestomach in mice is lined by stratified squamous epithelium that is contiguous with the esophagus up to the limiting ridge.

## 8. Profibrotic Mechanisms Aside of TGF-β

TGF-β signaling, with its downstream activation of Smad-mediated (also referred to as the canonical profibrotic pathway), [127] is the most widely involved mechanism in disease-associated fibrosis in many organs. However, non-canonical pathways have also been identified as primary drivers of this process in some organs and under specific circumstances. Non-TGF-β-mediated pathways involve different inflammatory agents and metabolic shifts in intercellular communication within the tissue microenvironment and can also trigger EMT; some of these pathways are presented below:

(a) Bone Morphogenetic Protein (BMP) is part of the TGF-β superfamily. BMP signaling regulates the maintenance of adult tissue homeostasis within multiple tissues [128], being mostly studied in bone. Its role in the morphogenesis of the digestive tract is increasingly recognized [129], however, including its role in the development of the Barrett esophagus [130]; but as of yet, no role in EoE has been described.
(b) Macrophages are highly heterogeneous cells of the innate immune system, involved in innate immunity, inflammatory responses, homeostasis, and tissue regeneration [131]. Th2 cytokines, such as IL-4 and IL-13, are able to: polarize macrophages to M2 cells, which produce chemokines that recruit Th2 lymphocytes and T regulatory; promote dysfunction, which can impair the proper regenerative process; and promote the development of fibrosis, deposition of type I and III collagen, and myofibroblasts activation [132]. The M2-derived specific chemokine CCL18 has been involved in pulmonary/alveolar, peritoneal, and hepatic fibrosis [133]. High plasma levels of CCL18 have been associated with progressive fibrosing disorders of the lungs and liver; and their elevated concentration in the peritoneal dialysis effluent predicts fibrosis and dysfunction of the peritoneal membrane [120]. Preliminary evidence points towards a role for M2 signaling in fibrosis associated with EoE [110]; and this should be further investigated.
(c) An IL-13-dependent fibrotic mechanism, independent of the downstream actions of TGF-β1 and MMP-9, was described in *Schistosoma mansoni* infection-related liver fibrosis [134]. These mechanisms might be particularly relevant for diseases where a vigorous type-2 cytokine response is also present, as in EoE, but no study has addressed them in this context to date.

## 9. Reversibility of Fibrosis in EoE

Fibrosis is a common outcome in many inflammatory diseases that may disintegrate the regular structure of organs and cause persistent damage. However, clinical observations and experimental models provide evidence that fibrosis is not irreversible, and once the chronic tissue injury is resolved, fibrosis may regress through the deactivation of myofibroblasts, degradation of ECM, and fibrolysis of excess matrix scaffold [52]. The ability of fibrosis to reverse and the degree of recovery may vary depending on the tissue type, its capacity to regenerate, its originating mechanism, and to a certain extent, on its degree of evolution. Termination of the underlying cause of tissue damage avoids further activation of myofibroblasts and deactivates the inflammatory pathways, while regenerative pathways in parenchymal cells are provided by the development of an anti-inflammatory microenvironment. Thus, myofibroblasts undergo apoptosis or revert to an inactive phenotype. The ECM is then degraded by matrix metalloproteinases (MMPs) that digest collagen and other components. Macrophages contribute to phagocytizing ECM fragments and to reducing MMP-inhibitory proteins [135].

Evidence of fibrosis reversion in EoE after effective anti-inflammatory therapy has been provided at both tissue and clinical levels [103,110,136,137]. The degree of lamina propria remodeling, demonstrated with Mason trichrome staining in esophageal biopsies obtained from children with EoE, before and after at least 3 months of therapy with budesonide, reduced fibrosis among responders, where TGF-β1- and pSmad2/3-positive cells were diminished [113]. Similar findings were reported in adults treated with fluticasone propionate for one year [110]. In this study, reduced fibrosis was visible in deep esophageal biopsies (including the whole lamina propria) accompanied by vanishing eosinophils in both epithelial and lamina propria layers and reduced mRNA levels for *IL-5*, *FGF-9*, and *CCL18*. Notably, mRNA expression levels of *TGF-β1* still remained. In another trial, treatment with viscous budesonide reduced the TGF-β epithelial cells by 75% in areas of intense eosinophil infiltration [109].

Rings and strictures are the endoscopic features characteristic of fibrosis in EoE, along with narrow caliber esophagi. Some evidence in the literature showed that endoscopic fibrotic changes in EoE may improve with anti-inflammatory therapy when assessed with endoFLIP. Improvements in esophageal dysmotility have been demonstrated with topical corticosteroids [138], diets [137,139], and even proton pump inhibitors [136]. Improvements in endoscopic features of fibrosis after anti-inflammatory treatment have also been described; recently, the benefit of Dupilumab (a monoclonal antibody that blocks the heterodimeric receptor common for IL-4 and IL-13) on severe, refractory, and fibrostenotic EoE was assessed. After a median of 6 months, most patients achieved histological, endoscopic, and symptom improvement, along with improved esophageal stricture diameter [140].

There are, however, few therapeutics that have shown to be effective once dense fibrotic tissue has been formed and patients present with strictures that cannot be passed with an endoscope. Endoscopic dilation has been used as a mechanical procedure to enlarge a narrowed esophageal lumen when patients are unable to swallow solid food properly or present repeated episodes of esophageal choking, which typically occurs with an esophageal diameter lower than 13 mm [141].

## 10. Conclusions and Perspectives

The recognition of EoE as a prevalent cause of esophageal symptoms, capable of deteriorating the quality of life of those who suffer from it, has been accompanied by rapid progress in the description of the cellular and molecular bases of the disease. The development of therapies for EoE now includes drugs specifically directed against cytokines, with a key role in the initiation and maintenance of the inflammatory response. Fibrous remodeling of the esophagus represents the main sequel of untreated or insufficiently treated disease, and the major consequence of delayed diagnosis. Collagen deposition in the esophagus, in its most advanced forms, seriously alters the structure and functionality of the esophagus and severely impairs quality of life. Limited evidence suggests that available anti-inflammatory treatments are effective in reversing fibrotic phenomena in EoE, at least in its initial stages, and preliminary experiences with new biological drugs seem promising for the most severe fibrotic forms of EoE. However, research on the molecular basis that leads to it has been scarce, and has often tried to reproduce in the esophagus mechanisms well known in other type 2 inflammatory diseases, such as bronchial asthma. Still, there is no solid evidence that fibrosis in EoE appears to be mainly mediated by TGF-β, thus opening the door to investigating alternative mechanisms largely independent of TGF-β. This approach might identify potential therapeutic targets directed to one of the most severe consequences of EoE.

## Figures and Tables

**Table 1:** Characteristics of the reviewed manuscripts.

Publication and Year	Species	Sample	Method	EoE Samples (n)	Outcome	Comparative Group	*p* Value
Aceves 2007 [103]	Human	Biopsies	IHC	7	Increased numbers of TGF-β1(+) cells in LP from EoE	GERD Healthy	*p* = 0.02 *p* = 0.004
Misrha 2008 [25]	Human Mice	Biopsies Tissue	RTqPCR	8 7	Increased expression level of TGF-β1 from EoE	Healthy WT mice	*p* < 0.001 *p* < 0.001
Aceves 2010 [107]	Human	Biopsies	IF	21	Increased numbers of TGF-β1(+) cells in SM from EoE	Healthy	*p* = 0.005
Aceves 2010 [113]	Human	Biopsies	IHC	16	Increased numbers of TGF-β1(+) cells in LP from EoE_R_	EoEpost_R_	*p* = 0.01
Dohil 2010 [108]	Human	Biopsies	IHC	15	Increased numbers of TGF-β1(+) cells in LP from EoEp	EoEpost	NA
Straumann 2010 [109]	Human	Biopsies	IF	18	Increased numbers of TGF-β1(+) cells from EoEpre	EoEpost	NA
Lucendo 2011 [110]	Human	Biopsies	RTqPCR	10	No significant increased expression levels of TGF-β1 from EoE	GERD Healthy EoEpost	*p* = 0.11
Kagalwalla 2012 [111]	Human	Biopsies	IHC	18	Increased score of TGF-β1 index of EoE, and correlate with EMT scores index (r = 0.520, *p* < 0.01)	GERD Healthy	*p* < 0.01 *p* < 0.001
Cho 2014 [105]	Mice	Tissue	IHC RTqPCR	12 (sensitized mice)	Increased numbers of TGF-β1(+) cells and expression levels in sensitized mice. No difference in numbers of TGFβ1 positive cells in sensitized mice Smad3 KO compared to sensitized WT mice (*p* = NS)	WT mice SMAD^−/−^ mice	*p* < 0.01 (IHC) *p* < 0.001 (qPCR)
Rieder 2015 [104]	Human	OCS	ELISA	14	Increased protein level of TGF-β1 from EoE	Healthy	*p* = 0.006
Collison 2015 [106]	Mice	Tissue	RTqPCR	NA	Increased expression level of TGF-β1 in sensitized mice.	WT mice	*p* < 0.05
Rajan 2015 [112]	Human	Biopsies	IHC	32	Increased numbers of TGF-β1(+) cells from EoE_R_ over time. (No alignment with fibrosis is found, except in the early stages of the disease course and only in responders)	EoE_R_ over time	*p* = NS
Rawson 2016 [114]	Human	Biopsies	IHC	14	Increased numbers of TGF-β1(+) cells in LP from EoE	Healthy	NA
Arias 2016 [115]	Human	Biopsies	RTqPCR	10	No significant increased expression levels of TGF-β1 from EoEpre	Healthy EoE post	*p* = 0.740 *p* = 0.386
Sarbinowska 2021 [117]	Human	Serum	ELISA	16	Increased protein level of TGF-β1 from EoE compared to controls but not significant differences to EoEpost.	Healthy EoE post	*p* = 0.04 *p* = 0.12
de Rooij 2022 [116]	Human	Biopsies	RTqPCR	40	Significant decreased expression levels of TGF-β1 from EoE after combined treatment, but no differences were observed after FFED treatment alone.	EoE post EoE post FFED	*p* < 0.01 *p* > 0.05
Pronio 2022 [118]	Human	Biopsies	RTqPCR	5	TGF-β expression was similar to the control in the mid-esophagus but reduced the distal EoE esophagus	Healthy	NA
Cao 2023 [119]	Mice	Tissue	WB	6	Compared with naïve mice, TGF-β1 protein levels in sham-treated mice increased and decreased significantly after treatment, comparing before and after treatment.	WT mice EoE mice post	*p* < 0.001 *p* < 0.01

Species: species of origin of the sample used in the study. Samples: type of samples used in the study. Method: technique used to measure TGF-β1 levels. Comparative groups: samples compared to those in the EoE group (GERD group/Healthy group or WT in mice). EoE samples (n): number of samples obtained from patients with EoE. Outcome: result of the statistical comparison of TGF-β1 levels. *p* value: statistical significance of comparative analysis. OSC: organ culture system. LP: lamina propria. SM: smooth muscle. IHC: immunohistochemistry. NA: not available; RTqPCR: quantitative reverse transcription polymerase chain reaction. ELISA: enzyme-linked immunosorbent assay. IF: immunofluorescence. GERD: gastroesophageal reflux disease. WT: wild type. EoER: responder patients to corticoids. EoENR: non-responders patients to corticoids. FFED: four-food elimination diet. EoEpost: EoE after effective treatment. EoEp: EoE placebo group/not treated. EoEpre: EoE before treatment, NS: not significant. Statistical significance *p* < 0.05.

## Data Availability

Not applicable.
